# The insect antimicrobial peptide cecropin A disrupts uropathogenic *Escherichia coli* biofilms

**DOI:** 10.1038/s41522-020-0116-3

**Published:** 2020-02-12

**Authors:** Miriam Kalsy, Miray Tonk, Martin Hardt, Ulrich Dobrindt, Agnieszka Zdybicka-Barabas, Malgorzata Cytrynska, Andreas Vilcinskas, Krishnendu Mukherjee

**Affiliations:** 1 Fraunhofer Institute for Molecular Biology and Applied Ecology, Department of Bioresources, 35394 Giessen, Germany; 2 Institute for Insect Biotechnology, Justus Liebig University, 35392 Giessen, Germany; 3 LOEWE Centre for Translational Biodiversity Genomics (LOEWE-TBG), 60325 Frankfurt, Germany; 4 Imaging Unit, Biomedical Research Center Seltersberg (BFS), Justus Liebig University, 35392 Giessen, Germany; 5 Institute of Hygiene, University of Muenster, 48149 Muenster, Germany; 6 Department of Immunobiology, Institute of Biological Sciences, Faculty of Biology and Biotechnology, Maria Curie-Skłodowska University, Lublin, Poland

**Keywords:** Biofilms, Antimicrobials

## Abstract

Current antibiotics cannot eradicate uropathogenic *Escherichia coli* (UPEC) biofilms, leading to recurrent urinary tract infections. Here, we show that the insect antimicrobial peptide cecropin A (CecA) can destroy planktonic and sessile biofilm-forming UPEC cells, either alone or when combined with the antibiotic nalidixic acid (NAL), synergistically clearing infection in vivo without off-target cytotoxicity. The multi-target mechanism of action involves outer membrane permeabilization followed by biofilm disruption triggered by the inhibition of efflux pump activity and interactions with extracellular and intracellular nucleic acids. These diverse targets ensure that resistance to the CecA + NAL combination emerges slowly. The antimicrobial mechanisms of CecA, thus, extend beyond pore-forming activity to include an unanticipated biofilm-eradication process, offering an alternative approach to combat antibiotic-resistant UPEC infections.

## Introduction

Uropathogenic *Escherichia coli* (UPEC) causes up to 90% of all urinary tract infections (UTIs) in humans, and antibiotics remain the primary therapeutic option despite the spread of resistance among UPEC strains.^1^ Treatment is challenging because UPEC can form biofilms in the bladder epithelial cells and also on the surface of urinary catheters.^2^ Biofilm-forming UPEC cells are highly resistant to current antibiotics because the doses have been optimized to kill planktonic cells.^3^ UPEC biofilms use multiple strategies to resist antibiotics, including extracellular appendages, an impenetrable extracellular matrix (ECM), persister cells, efflux pumps, and quorum sensing.^3^ Anti-biofilm drugs must overcome all these barriers and achieve broad-spectrum activity against both planktonic and biofilm-forming UPEC cells without adverse effects.

Antimicrobial peptides (AMPs) are promising candidates for biofilm eradication given their novel mechanisms of action against planktonic cells, which reduce the likelihood of resistance evolving compared to the use of antibiotics.^4^ Furthermore, combinations of AMPs and traditional antibiotics with different mechanisms of action could facilitate the revival of ineffective drugs based on the enhanced or synergistic activity of the combination against human pathogens.^5^ However, most AMPs have not been tested to determine their ability to inhibit biofilm formation by UPEC strains, and careful re-screening is therefore necessary to identify promising candidates.

In this study, we screened insect AMPs for anti-biofilm activities and found that cecropin A (CecA) from the greater wax moth *Galleria mellonella* can eradicate biofilms formed by UPEC strains. We tested combinations of this AMP with the antibiotic nalidixic acid (NAL) in vitro and in vivo to determine the potential for synergistic interactions. We also investigated the ability of CecA to interact with multiple extracellular and intracellular targets that are important for anti-biofilm drug discovery.

## Results and discussion

### Testing the synergistic antibacterial activity of CecA and NAL against UPEC cells

**Fig. 1:**
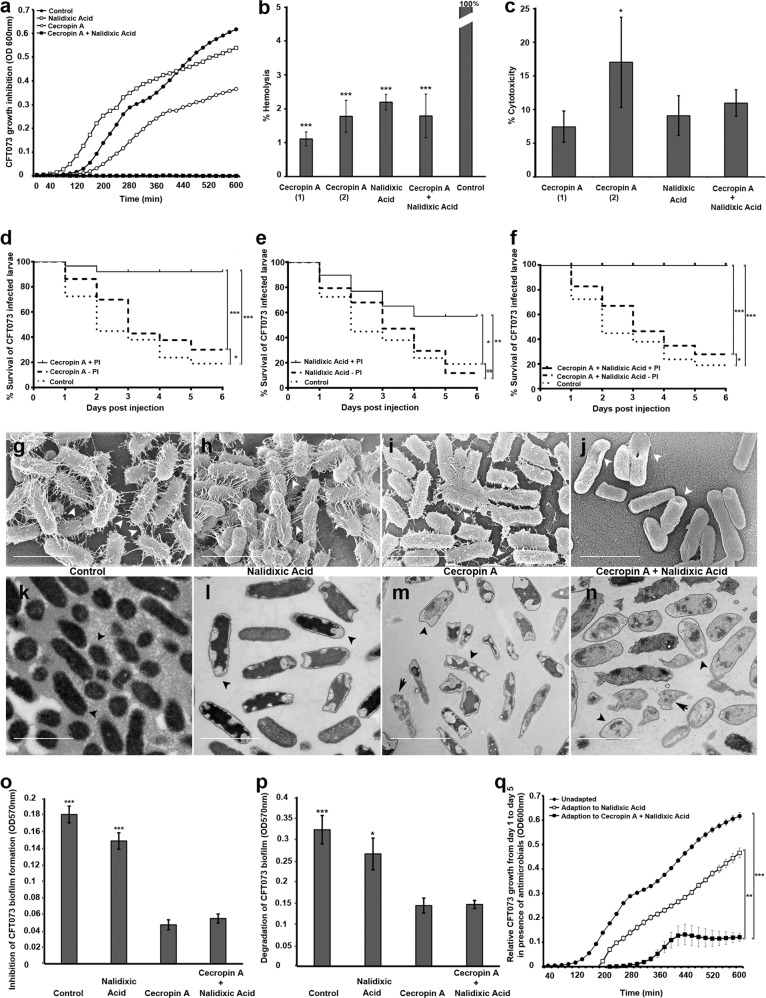
Effect of synergy between cecropin A (CecA) and nalidixic acid (NAL) on planktonic and biofilm-forming uropathogenic *Escherichia coli* cells. **a** Time-kill curves of CecA (50 μg/ml), NAL (0.5 ng/ml), and their combination against strain CFT073. Cells were treated with CecA 1 (50 μg/ml), CecA 2 (75 μg/ml), NAL (0.5 ng/ml), or CecA (50 μg/ml) + NAL (0.5 ng/ml) to determine **b** hemolysis of porcine erythrocytes in comparison to 10% Triton X-100 (control), and **c** the viability of BHK-21 fibroblast in comparison to untreated control. Kaplan-Meier plots of CFT073-infected *G. mellonella* survival after treatment with **d** CecA (50 μg/ml), **e** NAL (0.5 ng/ml), and **f** their combination +/− protease inhibitor (PI). **g–j** Scanning and **k**–**n** transmission electronic microscopy analysis of biofilm structures formed by CFT073 following treatment with CecA (50 μg/ml) ± NAL (0.5 ng/ml). **g–n** The biofilm-containing discs were supplemented with **h**, **l** NAL, **i**, **m** CecA, or **j**, **n** their combination in fresh LB medium and incubated for another 24 h. Biofilm-forming discs grown in LB medium without supplements were used as controls (**g**, **k**). Representative images are shown (scale bar = 2 μm). White arrow heads show intercellular filaments (**g–j**), black short arrow heads indicate cytoplasm density (**k**–**n**), and black arrows show cell debris (**m, n**). **o** Percentage inhibition of CFT073 biofilms by NAL (0.5 ng/ml) or CecA (50 μg/ml) compared to their combination after 48 h. **p** Percentage eradication of pre-formed CFT073 biofilms by NAL (0.5 ng/ml) or CecA (50 μg/ml) compared to their combination. **q** CFT073 adaptation to NAL (20 μg/ml) or the CecA (20 μg/ml) + NAL (0.5 ng/ml) combination was determined by analyzing bacterial growth after repeated exposure to antimicrobials for 5 days. The figure represents bacterial growth at day 5 with reference to day 1 after antimicrobial exposure. Values are means with standard errors: *n* = 4 (panels **a** and **q**), *n* = 3 (panels **b–f** and **o–p**). Significance was determined by one-way ANOVA, Dunnett’s multiple comparison test (**b**, **c**, **o**, **p**), Holm-Šídák correction (**q**) (*P < 0.05; **P < 0.005; ***P < 0.0005).

When screening insect AMPs against UPEC strains, we discovered the “biofilm-busting” activity of CecA, a linear cationic α-helical peptide from the greater wax moth *Galleria mellonella*. The minimum inhibitory concentration (MIC) of CecA against the planktonic UPEC strain CFT073 was high, so we tested combinations of this AMP with the antibiotic nalidixic acid (NAL) in a checkerboard assay to identify synergistic interactions. The absolute MIC decreased from 100 to 50 μg/ml for CecA, and from 60 μg/ml to 0.5 ng/ml for NAL, indicating their synergistic activity (Fig. 1a and Supplementary Fig. 1). This was confirmed by calculating the fractional inhibitory concentration (FIC < 0.5). We selected the combination of 50 μg/ml CecA and 0.5 ng/ml NAL for further analysis because it is the minimum CecA and NAL concentration, respectively, that is ineffective as monotherapy but can synergistically interact to completely inhibit the growth of planktonic UPEC cells. The antimicrobial concentrations of CecA (50 μg/ml), NAL (0.5 ng/ml) and their combination showed no significant toxicity toward erythrocytes (CecA < 1.5%, NAL < 2.5%, CecA + NAL < 2.5%) or fibroblasts (CecA < 8%, NAL < 10%, CecA + NAL < 11%), suggesting that CecA is suitable for further therapeutic development (Fig. 1b, c). However, cytotoxicity was slightly increased with 75 μg/ml of CecA (Fig. 1c). We therefore investigated the efficacy of CecA in vivo using our *G. mellonella*–UPEC infection model^6^ with or without NAL (Fig. 1d–f). *G. mellonella* is an ideal replacement for rodents and other mammalian infection models because the larvae can be reared at 37 °C and are susceptible to many different human pathogens, but large numbers of insects can be reared and tested inexpensively without ethical restrictions, providing extremely robust and statistically significant in vivo data.^6^ CecA ± NAL slightly improved the survival of CFT073-infected larvae but NAL alone had no effect (Fig. 1d–f). CFT073 survival in infected larvae was reduced more significantly by the combination of 50 μg/ml CecA and 0.5 ng/ml NAL compared to individual treatments (Supplementary Fig. 2).

AMPs are proteolytically degraded in vivo so we added a protease inhibitor to improve the therapeutic efficacy of CecA.^7^ Toxicity of fibroblast cells was slightly increased by the CecA + NAL combination in presence of the protease inhibitor (Supplementary Fig. 3a, b). We found that more of the infected larvae survived when the protease inhibitor was present (Fig. 1d–f). We then analyzed the expression of genes regulating phases I and III of drug metabolism in *G. mellonella* larvae to determine whether the protease inhibitor affected the pharmacokinetic properties of the combined treatment in vivo (Supplementary Table 1). We selected genes encoding cytochrome P450, alcohol dehydrogenase, NADH-ubiquinone oxidoreductase, carboxylic ester hydrolase, epoxide hydrolase (phase I), and the efflux transporter ATP-binding cassette proteins (phase III) that are responsible for drug metabolism. These genes were significantly upregulated in infected larvae 1 h following a single dose of the combined treatment compared to the AMP without the protease inhibitor (Supplementary Fig. 3c, d). This indicated increased metabolic activities in larvae possibly due to sustained therapeutic efficacy of CecA because of the presence of the protease inhibitor. Furthermore, the protease inhibitor could inhibit protease activity in *G. mellonella* hemolymph by >50% (Supplementary Fig. 3e). Based on these pharmacokinetic parameters, we selected 50 μg/ml CecA and 0.5 ng/ml NAL as the most suitable dose to investigate the viability and membrane integrity of planktonic and biofilm-forming CFT073 cells.

### Effects of CecA on the viability and structure of biofilm-forming UPEC cells

We determined the viability of cells using the vital stain SYTO 9, which binds to living cells with intact membranes and stains them green, and propidium iodide, which specifically penetrates cells with damaged membranes and stains them red. Planktonic CFT073 cells treated with dimethyl sulfoxide as a control were stained green but the proportion of red cells increased progressively following treatment with NAL, CecA, and CecA + NAL, respectively (Supplementary Fig. 4a–d). Bacterial surface was less granular with damaged envelopes and recesses following CecA treatment indicating loss of membrane integrity in comparison to untreated *E. coli* cells (Supplementary Fig. 5 and Supplementary Table 2). We observed up to 15% permeabilization of the bacterial membrane by CecA at the final concentration range of 0.25 µM–1 µM (Supplementary Fig. 5a–e). Cecropins in contrast to NAL permeabilize bacteria by initial binding to outer membrane components like lipopolysaccharides (LPS)^5^ in Gram-negatives, and accordingly, we found that the combination of CecA + NAL caused a significant increase in cell permeability, reflecting their synergistic activity.

Electron microscopy revealed that CecA ± NAL prevents the development of UPEC biofilms by targeting UPEC adhesive filaments, specifically type I and P fimbriae (Fig. 1g–j), that facilitate the permanent attachment of biofilm-forming cells to surfaces and limit contact between AMPs and bacterial membranes.^3^ Control and NAL-treated CFT073 biofilms were visually indistinguishable, featuring complex networks of rod-shaped cells (Fig. 1g, h). However, treatment with CecA ± NAL destroyed this network, resulting in the collapse and subsequent disruption of the bacterial membrane (Fig. 1i, j). These treatments also caused changes in the structure of the polymeric ECM, cell envelope, and cytoplasmic density of the UPEC cells compared to controls (Fig. 1k–n). The severity of the damage was greatest for the CecA ± NAL treatment, and included an irregular shape, damaged outer membranes, large sites of cytoplasmic clearing, and cell debris (Fig. 1m, n). These data indicate that CecA ± NAL inhibits planktonic cells of different UPEC strains (CFT073 and 536) and also degrades surface-associated biofilms of these strains, probably by enhancing the diffusion of NAL into the cell (Fig. 1o, p and Supplementary Fig. 6a–f). This is likely to limit the opportunity for UPEC cells to evolve resistance against CecA, which forms pores in the bacterial membrane, as this would require simultaneous alterations to multiple biochemical pathways related to cell membrane modifications.^8,9^

### UPEC adaptation to the combination of CecA + NAL

We next considered whether UPEC can adapt to the combined drug treatment and tested this by exposing CFT073 cells to sublethal concentrations of CecA (20 μg/ml) and NAL (0.5 ng/ml) repeatedly for 5 days. We then compared the adapted UPEC population (day 5) to the ancestral population (day 1) by exposing them both the same combination dose (Fig. 1q). A similar strategy was followed with sublethal concentrations of NAL alone (20 µg/ml). Repeated exposure to the combination of CecA + NAL led to a significant decline in bacterial fitness, as shown by the slow recovery of growth on day 5 compared to cells treated with NAL alone. This indicated that CFT073 cells take longer to evolve resistance against the CecA + NAL combination than to the antibiotic alone.^4^ Future research should address whether the delayed resistance to the CecA + NAL combination is mediated by CecA targeting a single or multiple bacterial components.

### Identification of extracellular and intracellular targets of CecA in biofilm-forming UPEC cells

**Fig. 2:**
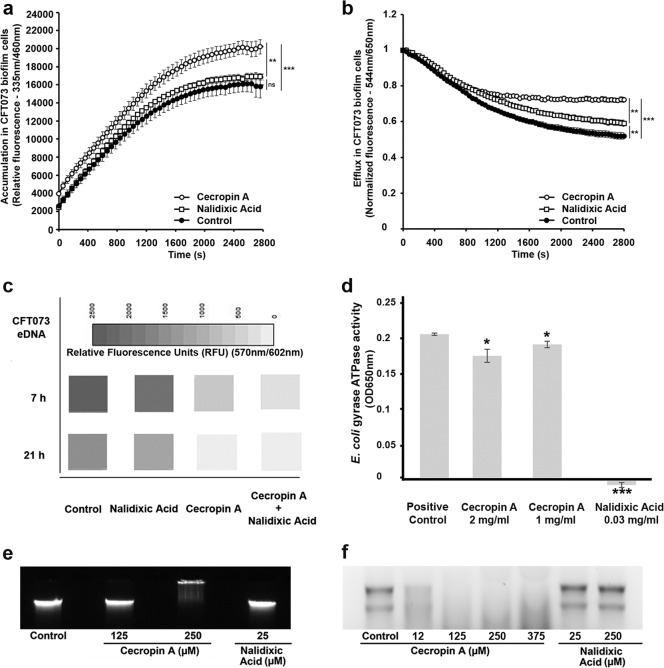
Molecular targets of cecropin A (CecA) in uropathogenic *Escherichia coli* cells. **a** Steady-state levels of H33342 (2.5 µM) accumulating in CFT073 biofilm cells with and without exposure to CecA (10 µg/ml) or nalidixic acid (NAL; 0.5 ng/ml). **b** Inhibition of Nile red efflux by CecA (10 µg/ml) and NAL (0.5 ng/ml). Efflux was triggered at 100 s by the addition of 20 mM glucose. The intensity of fluorescence emission from Nile red was presented to show the effects of CecA, NAL or the absence of treatment in CFT073 biofilm cells. **c** Decreasing intensities of the fluorescent dye BOBO-3 that cannot penetrate intact membranes and consequently only stains external nucleic acids were measured following incubation with untreated CFT073 cells or those treated with NAL (0.5 ng/ml), CecA (50 μg/ml) or their combination. **d** Inhibition of *E. coli* DNA gyrase ATPase activity by CecA and NAL. Decreasing intensities of eDNA and **f** RNA bands from CFT073 cells treated with a high concentration of CecA were visualized by gel electrophoresis to highlight the DNA/RNA-binding activity of CecA. Values are means and standard errors: *n* = 3 (panels **a**, **b**, and **d**). Significance was determined by one-way ANOVA and Holm-Šídák correction (**a**, **b**), Dunnett’s multiple comparison test (**d**) (*P < 0.05; **P < 0.005; ***P < 0.0005; ns—not significant).

The antimicrobial resistance of bacterial biofilms is mediated by multiple factors including energy-dependent multidrug resistance efflux pumps and extracellular DNA (eDNA), a component of the ECM.^10–13^ We used fluorescent dyes with different emission wavelengths depending on their location (intracellular or extracellular) to measure the accumulation, efflux and eDNA-binding capacity of CecA in CFT073 cells.^14,15^ Non-inhibitory antimicrobial concentrations were selected to study the accumulation of the peptide and the efflux activity, thus preventing changes in fluorescence intensity due to the loss of membrane integrity. Biofilm- forming UPEC cells were incubated with 10 µg/ml CecA or 0.5 ng/ml NAL in the presence of 2.5 µM H33342, a DNA-intercalating fluorescent dye. Similarly, we incubated exponential-phase CFT073 cells (planktonic cells) with 1 µg/ml CecA or 0.5 ng/ml NAL in the presence of 2.5 µM H33342. CecA caused a significant increase in H33342 accumulation by both planktonic cells and UPEC biofilm compared to NAL and the untreated control (Fig. 2a and Supplementary Fig. 7a). We also measured the efflux of the lipophilic dye Nile red, which emits a stronger signal when bound to membrane phospholipids and is therefore ideal for the investigation of resistance-nodulation-division (RND) efflux pumps.^14^ We detected a stronger fluorescent signal in the presence of CecA compared to NAL and the untreated control, indicating that dye efflux was inhibited by CecA (Fig. 2b and Supplementary Fig. 7b). Dye efflux was inhibited in biofilm and planktonic UPEC cells in presence of 10 μg/ml and 1 μg/ml CecA, respectively (Fig. 2b and Supplementary Fig. 7b, c). Similarly, we stained CFT073 biofilms with BOBO-3, which selectively binds eDNA, and screened for changes in fluorescence caused by dye displacement. We observed a decrease in fluorescence intensity when CFT073 was incubated with 50 μg/ml CecA ± 0.5 ng/ml NAL for 7 or 21 h, indicating that CecA inhibits the release of eDNA. In contrast, NAL alone had no effect on the signal and thus does not appear to influence the release of bacterial eDNA (Fig. 2c). These data confirm that the degradation of UPEC biofilms is promoted by the suppression of efflux, the uptake of more CecA, and its ability to bind eDNA.

Next, we investigated the interaction between CecA and other UPEC intracellular targets (genomic DNA and total RNA). *E. coli* DNA gyrase ATPase activity, which is required for DNA supercoiling and decatenation, was inhibited strongly by NAL but only weakly by CecA (Fig. 2d). Using the limbo server, we predicted that CecA would bind to the WKIFKKI sequence of DnaK (score = 13.4), but we found no evidence that the peptide inhibits the ATPase activity of DnaK (Supplementary Fig. 7d).^16^ We also predicted the presence of DNA/RNA-binding residues in CecA using DP-Bind and FastRNABindR (Supplementary Tables 3 and 4). We observed the concentration-dependent displacement of DNA (Fig. 2e and Supplementary Fig. 8) and RNA (Fig. 2f and Supplementary Fig. 9) by high doses of CecA. Taken together, our data show that CecA is a biofilm-busting AMP and that its multi-target mechanisms of action make it suitable as a lead for the development of new drugs that eradicate biofilm-forming UPEC strains.

## Methods

### Peptides, bacterial strains, and culture conditions

CecA is 39 amino acids long (KWKIFKKIEKAGRNIRDGIIKAGPAVSVVGEAATIYKTG) and was synthesized by Pepmic, Jiangsu, China. NAL was sourced from Sigma-Aldrich (St. Louis, Missouri, USA). We tested the UPEC strains CFT073 (pyelonephritis isolate) and 536 (cystitis isolate) that were susceptible to all clinically used antibiotics. Bacterial cultures were maintained aerobically in lysogeny broth (LB) medium (Carl Roth, Karlsruhe, Germany) at 37 °C and on LB agar plates. For long-term storage, bacteria were frozen at –80 °C in LB medium supplemented with 30% glycerol.

### Inhibition of bacterial growth

Logarithmic growth phase bacterial cultures in 10 ml LB were diluted to a final OD_600_ of ~0.002 and MICs were determined using the broth microdilution method in 96-well microtiter plates with an assay volume of 200 µl. Briefly, serial dilutions of NAL (final concentration 60–0.0005 µg/ml) and CecA (final concentration 0.5–100 µg/ml) were transferred to the microtiter plate and incubated overnight at 37 °C. We then measured the absorbance at 600 nm to determine growth inhibition as an indicator of antimicrobial activity. The MIC was defined as the minimum concentration at which no bacterial growth was observed in each well.

### Checkerboard assay

The synergy between CecA and NAL was tested using a checkerboard assay.^17^ Bacterial suspensions were prepared at a final concentration of 5 × 10^5^ CFU/ml. The two compounds for combinatorial testing were arrayed as serial dilutions, vertically for one compound and horizontally for the other, in the same 96-well microplate. The assay was carried out in triplicate as described above for the measurement of individual MICs. The FIC indices were calculated according to the following formulae: FIC = *X*/MICX, and FIC index = FIC_CecA_ + FIC_NAL_, where *X* is the lowest inhibitory concentration of the drug in the presence of the co-drug. A FIC index of <0.5 indicates synergy between the test compounds.^18^

### Time-kill curve

A time-kill assay was carried out in triplicate, in non-cation-adjusted LB according to the protocol from the Clinical and Laboratory Standards Institute (Wayne, Pennsylvania, USA). An overnight bacterial culture was diluted in LB medium to an absorbance of 0.05 at 600 nm. Different concentrations of test compounds (CecA, NAL, or CecA + NAL) were then added, and the culture was incubated at 37 °C with shaking.

### Hemolytic and cytotoxicity assay

The hemolytic activities of the test compounds were determined with assay volumes of 100 μl.^19^ Erythrocytes were harvested by centrifugation at 1500 × *g* for 3 min at 4 °C and washed three times in phosphate-buffered saline (PBS). A suspension of the erythrocytes was prepared with a dilution factor of 1:10 in PBS. Test compounds with or without a broad-spectrum protease inhibitor cocktail (final concentration 0.0007×) (Sigma-Aldrich) that selectively inhibits cysteine and serine proteases but not metalloproteases or aspartic proteases, were incubated with the erythrocyte suspension for 1 h at 37 °C in a 96-well plate. Percentage hemolysis of the test compounds was calculated in comparison to 10% Triton X-100 (positive control).

Cytotoxicity was evaluated using the baby hamster kidney fibroblast cell line BHK-21 (Chromotek, Planegg-Martinsried, Germany).^20^ The cells were grown in the presence of Dulbecco’s modified Eagle’s medium (DMEM) supplemented with 4.5 g/l glucose, 110 mg/l sodium pyruvate and L-glutamine, and 10% fetal bovine serum (FBS). The cells were maintained in an NU-5810 incubator (IBS Tecnomara, Fernwald, Germany) at 37 °C with a 5% CO_2_ atmosphere. The cells were then sub-cultured at ~90% confluence by detaching with 0.25% trypsin and 0.03% EDTA (Sigma-Aldrich). Test compounds with or without the protease inhibitor (final concentration 0.0007×) were dissolved in water and diluted in DMEM. One day before each experiment, cells were plated at a density of 8 × 10^4^ cells/ml in Cell Star 96-well plates (Greiner Bio-One, Frickenhausen, Germany). The cells were rinsed with PBS before adding 100 µl of the test compound and incubating for 3.5 h at 37 °C. Test compounds were then removed and the cells were rinsed with PBS before staining with 10% v/v AlamarBlue (Bio-Rad, Puchheim, Germany) in DMEM for 2 h. The fluorescence was measured using an Eon microplate reader (BioTek, Winooski, Vermont, USA) using excitation and emission wavelengths of 528 and 590 nm, respectively. Cells exposed to DMEM without test compounds were used as a negative control and DMEM only was used as a blank reference. Percentage cytotoxicity of the test compounds was calculated in comparison to the negative control.

### UPEC—*G. mellonella* infection model and protease assay

We analyzed the survival of UPEC strain CFT073 in infected *G. mellonella* larvae.^6^ Briefly, larvae were homogenized in LB medium 1 h post injection and plated onto *E. coli* selective “*E. coli* direct” (ECD) agar (Sigma-Aldrich). The plates were incubated at 37 °C for 24 h and the colonies were counted. To confirm the presence of *E. coli* colonies, an indole test was performed using Kovac’s reagent (Sigma-Aldrich) according to the manufacturer’s protocol.

For the analysis of infected larvae survival, logarithmic UPEC CFT073 cultures with a density of 10^9 ^CFU/ml in 10 ml LB medium were washed and serially diluted in 0.9% NaCl to appropriate concentrations. To calculate the CFU of the inoculum, 50 µl of the injection dilution was plated on LB agar and incubated at 37 °C for 24 h. We then injected 10 µl of the bacterial inoculum (10^6^ CFU/ml) dorsolaterally into the hemocoel of last-instar larvae.^21^ After incubating at 37 °C for 30 min, we injected the test compounds with or without the protease inhibitor. Larvae were considered dead when they showed no tactile response after incubation at 37 °C for 5 days.

Last-instar larvae were injected with 10 µl of the protease inhibitor cocktail described above and were incubated at room temperature for 1 or 3 h before pricking the integument and withdrawing 100 µl of hemolymph. The hemolymph was centrifuged at 870 × *g* for 10 min at 4 °C. The supernatant was transferred to a black 96-well plate on ice. A Pierce Fluorescence Protease Assay Kit (Thermo Fisher Scientific) was then used to measure protease activity according to the manufacturer’s instructions. The intensity of fluorescein fluorescence was measured using a monochromator-based microplate reader (BioTek) with excitation and emission wavelengths of 485 and 538 nm, respectively. The work involving invertebrates such as *G. mellonella* does not need ethical permission according to German law.

### Reverse transcription polymerase chain reaction (RT-PCR) analysis of metabolic gene expression

Complementary DNA was synthesized using the First-strand complementary DNA (cDNA) Synthesis Kit (Thermo Fisher Scientific). The cDNA concentration was determined by spectrophotometry. Quantitative real-time RT-PCR was performed using the CFX 96 real-time PCR system (Bio-Rad Laboratories, Hercules, California, USA) and a SensiMix SYBR No-ROX kit (Bioline, Luckenwalde, Germany). We used 50 ng of cDNA per reaction to amplify gene sequences related to drug metabolism phases I and III using the primers shown in Supplementary Table 5. The amplification parameters comprised an initial activation step at 95 °C for 10 min, followed by 39 cycles of denaturation at 95 °C for 15 s, annealing at 56 °C for 15 s and extension at 72 °C for 15 s. The relative expression levels of the target genes were determined using the ΔΔCT method and the gene encoding 18S ribosomal RNA for data normalization.

### Live and dead staining of bacteria exposed to antimicrobial compounds

UPEC strain CFT073 was cultured at 37 °C until the cells reached the logarithmic growth phase. The cells were harvested and washed in PBS, and then a suspension of ∼10^3^ CFU/ml was treated with an equal volume of CecA (50 μg/ml), NAL (0.5 ng/ml), a combination (50 μg/ml CecA + 0.5 ng/ml NAL), or the negative control (1% PBS). After incubating at 37 °C for 3 h, the cultures were harvested and stained with 0.3% SYTO 9 and propidium iodide at ratio of 1:1 from the Live/Dead BacLight bacterial viability and counting kit (Thermo Fisher Scientific, Waltham, Massachusetts, USA). After mixing, the samples were mounted on microscope slides and observed using a Leica fluorescent microscope with diode lasers (excitation and emission wavelengths 488 and 561 nm, respectively).

### Bacterial membrane permeabilization assay

The membrane permeabilizing activity of the peptides was determined on the basis of a β-galactosidase activity level using the *E. coli* JM83 cells bearing plasmid pCH110 (Pharmacia-Amersham, Piscatway, NJ, USA), encoding constitutively synthesized cytoplasmic β-galactosidase.^22^ Briefly, the peptide was pre-incubated for 15 min at 37 °C in 20 mM phosphate buffer pH 6.8 (23 μl). Then, 2 μl of mid-logarithmic phase *E. coli* cells suspension (5 × 10^5^ CFU) in the same buffer were added. After 45 min incubation at 37 °C 20 mM HEPES/150 mM NaCl buffer pH 7.5 (220 μl) and 50 mM aqueous solution of *p*-nitrophenyl-β-D-galactopyranoside (5 μl) were added. The absorbance proportional to the β-galactosidase activity was measured at 405 nm after 1.5 h incubation at 37 °C. Live bacteria incubated alone and dead bacteria after treatment with 5 µM synthetic cecropin B (Sigma-Aldrich) served as the control samples. The permeabilization level of the dead bacteria was assumed as 100%. All assays were performed in triplicate in six independent experiments.

### Atomic force microscopy (AFM) imaging

Log-phase *E. coli* JM83 suspension (100 μl) (OD_600_ = 0.2) in the LB were incubated for 1.5 h at 37 °C without (control) and in the presence of the CecA (the final concentrations 0.25 μM).^22,23^ Next, the samples were centrifuged at 8000 × *g* for 10 min at 4 °C and washed twice with non-pyrogenic water. After final centrifugation, the bacteria were suspended in non-pyrogenic water (5 μl), applied on mica disks, and the samples were allowed to dry overnight at 28 °C.

Bacterial cell surface was imaged using NanoScope V AFM (Veeco, USA). A ScanAsyst-HR operation mode was used for topography imaging, as well as for the roughness and adhesion analyses measurements, whereas for Young’s modulus analysis the PeakForce QNM operation mode was applied. All measurements were done using a RTESPA silicon tip with a spring constant of 20 N/m (Bruker, Germany). The data were analyzed with Nanoscope Analysis ver. 1.40 software (Veeco, USA). Three fields on each mica disk were imaged. The Young’s modulus values were calculated from five 500 × 500 nm images. The average surface root-mean-square (RMS) roughness and adhesion values were calculated from sixty fields (80 × 80 nm) measured over the entire bacterial cell surface on 500 nm × 500 nm areas. Three dimensional images and section profiles were done using WSxM 5.0 software.^23^

### Determination of degradation/inhibition of UPEC biofilms by crystal violet assay

Logarithmic bacterial cultures were cultivated in LB medium supplemented with 0.8% glucose at 37 °C, without shaking, in 96-well plates (∼5 × 10^7^ cells/well) to initiate biofilm formation. Different strategies were adopted for analyzing the inhibition and degradation of UPEC biofilms in the presence of test compounds. The inhibition of biofilm formation was assayed by incubating UPEC strain CFT073 and 536 with test compounds (CecA, NAL, or CecA + NAL) for 48 h. To access biofilm degradation, UPEC was cultivated for 18 h to initiate biofilm formation. The culture medium and non-bound bacteria were removed by washing three times with sterile water, and attached cells were resuspended in LB medium supplemented with 0.8% glucose and the test compounds (CecA, NAL, or CecA + NAL) and incubated for another 18 h. The culture medium was discarded, and the wells were washed three times with sterile water before staining with 0.1% crystal violet for 30 min. Excess stain was removed by three washes with sterile water. The biofilm-bound crystal violet was solubilized by adding 130 μl 30% acetic acid and quantified by spectrophotometry at 570 nm.

### Determination of viable numbers of UPEC biofilm cells by plate count

UPEC strains CFT073 and 536 were cultivated in LB medium supplemented with 0.8% glucose and incubated with the test compounds (CecA, NAL, or CecA + NAL) for 24 h without shaking. The biofilm-forming UPEC cells with and without the antimicrobials were suspended and homogenized in LB via scraping and vortexing. Serial dilutions of the bacterial cultures were plated onto LB agar, and CFUs were counted after 24 h incubation.

### Electron microscopy

For transmission electron microscopy (TEM), test and control groups of bacteria were fixed with 3% formaldehyde and 0.5% glutaraldehyde in 0.1 M cacodylate buffer, embedded in gelatin (Sigma-Aldrich), post-fixed in 1.5% osmium tetroxide in the same buffer, washed with the buffer and incubated in 1% aqueous uranyl acetate (PolySciences, Niles, Illinois, USA) overnight at 4 °C. Specimens were dehydrated in an ethanol series (30, 50, 70, 80, 90, 96, 100% (v/v), 20 min each) and embedded in Epon (Serva Electrophoresis, Heidelberg, Germany). After heat curing, sliver to gold ultrathin sections were cut and contrasted in uranyl acetate and lead citrate. Ultrathin sections were visualized using an EM912a/b TEM (Carl Zeiss, Oberkochen, Germany) at 120 kV under zero-loss conditions and images were recorded with slight underfocus using a cooled 2k × 2k slow-scan charge-coupled device camera and the iTEM package (Olympus Soft Imaging Solutions, Münster, Germany).^24^

For scanning electron microscopy (SEM) analysis of bacterial surface, cells were grown on discs and after test and control treatment the specimens were fixed, post-fixed, washed, and dehydrated in an ethanol series as above. After critical point drying, the cells were mounted on SEM holders and sputtered with gold. Samples were observed in a DSM982 field emission gun scanning electron microscope (Carl Zeiss) at 3–5 kV. Images were recorded using a secondary electron detector with the voltage of the collector grid biased to +300 V in order to improve the signal-to-noise ratio and achieve an optimal topographical contrast.^24^

### Bacterial adaptation to antimicrobial compounds

The ability of UPEC CFT073 cells to adapt to NAL or the NAL + CecA combination was tested using the microdilution method described above. Cells at an OD_600_ of ~0.002 were exposed to a sub-inhibitory concentration of NAL (20 µg/ml) for 24 h, then they were diluted to OD_600_ ~0.002 in fresh LB medium supplemented with the same concentration of NAL for a further 24 h. This process of serial passaging and exposure was continued for 5 days. Similarly, cells were exposed to a sub-inhibitory concentration of the combination of NAL (0.5 ng/ml) and CecA (20 µg/ml) for 5 days. Absorbance was measured at 600 nm to determine growth.

### Accumulation assay

UPEC strain CFT073 was used to develop and evaluate an assay based on the intracellular accumulation of the fluorescent probe H33342 bisbenzimide (Sigma-Aldrich).^12^ The toxicity of H33342 (2.5 µM) was determined by the incubation of CFT073 at a mid-log growth phase OD_600_ of 0.6 for 24 h, and bacterial growth was determined using a plate reader (BioTek).

Planktonic bacterial strains were grown at an exponential growth phase OD_600_ of 0.6 (=10^8^ CFU/ml) with aeration in LB at 37 °C. Bacteria were also cultivated for 24 h in LB medium supplemented with 0.8% glucose at 37 °C, without shaking to initiate biofilm formation. UPEC biofilms were suspended and homogenized in LB via scraping and vortexing. Planktonic and biofilm cells were harvested by centrifugation at 2100 × *g* for 2 min at room temperature and resuspended in 1 ml PBS. The OD_600_ of the suspension was measured and adjusted to 0.5 to standardize the number of cells in each culture and to simulate the conditions used in the H33342 accumulation assay. A 180-µl aliquot of bacterial culture was supplemented with CecA (1 µg/ml for planktonic cells and 10 µg/ml for biofilm cells) or NAL (0.5 ng/ml) and 20 µL H33342 (2.5 µM) in the wells of a black microtiter plate, with PBS as a negative control. Fluorescence intensity was measured (excitation and emission wavelengths of 335 and 460 nm, respectively) after incubating at 37 °C. Raw fluorescence values were analyzed using Microsoft Excel (Microsoft, Redmond, Washington, USA), including the calculation of mean values for each column and the subtraction of blanks.

### Efflux assay

An overnight culture of the UPEC strain was diluted and grown to exponential phase (planktonic cells). Similarly, an overnight culture of the UPEC strain was diluted and grown in LB medium supplemented with 0.8% glucose at 37 °C for 24 h, without shaking to initiate biofilm formation. UPEC biofilms were collected in LB via scraping and vortexing. Aliquots of the culture were centrifuged at 2100 × *g* for 10 min at room temperature. The pellet was resuspended in 20 mM PBS (pH 7.4) containing 1 mM MgCl_2_. After another centrifugation and resuspension step as above, the cells were adjusted to an OD_600_ of 1.0 in PBS. All subsequent operations were carried out at room temperature, except as noted. The cells were allowed to rest for ~20 min, and 500-µl aliquots were then transferred to Pyrex 15-ml conical centrifugation tubes (glass tubes were used because Nile red is adsorbed to the walls of plastic tubes). Aliquots of this culture were supplemented with CecA (1 µg/ml for planktonic cells and 10 µg/ml for biofilm cells) or NAL (0.5 ng/ml) in glass test tubes and incubated at 37 °C for 20 min. Untreated CFT073 cells were used as a negative control. After 20 min, Nile red stock solution (1 mM in 9:1 (v/v) ethanol:dimethylformamide) was added to a final concentration of 1 μM, and the cell suspension was incubated at 37 °C for 1 h. After centrifugation at 2100 × *g* for 5 min at room temperature, most of the supernatant was discarded, and droplets clinging to the tube wall were removed using absorbent tissues. The cells were resuspended in 2 ml PBS and fluorescence was measured using a microplate reader (BioTek) at excitation and emission wavelengths of 544 and 650 nm, respectively, with and without glucose (20 mM), which triggers efflux in bacteria. We analyzed Nile red efflux at three glucose concentrations (50, 20, and 10 mM) and selected 20 mM for meaningful quantification of UPEC efflux potential (Supplementary Fig. 7c).

Data from experiments for normalization of dye fluorescence (*y*-axis) versus time (*x*-axis) were plotted using GraphPad Prism v7.05 (GraphPad Software, La Jolla, California, USA). The Nile red and treated curves were fitted using a single exponential decay (one phase decay) equation:

$$Y = \left( {Y_0 - \mathrm{plateau}} \right) \times {\mathrm{exp}}\,\left( { - K \times X} \right) + \mathrm{plateau}$$

where *X* is the time, and *Y* starts at *Y*_0_ and then decays in one phase down to plateau. The plateau is the *Y* value at infinite time. The span is the difference between *Y*_0_ and the plateau and has the same units as *Y*. *K* is the rate constant.^25^

### DNA/RNA/eDNA-binding, gyrase, and DnaK ATPase activity assays

UPEC strain CFT073 genomic DNA/total RNA was extracted using the bacterial genomic DNA extraction kit (Sigma-Aldrich) and RNeasy Mini Kit (Qiagen, Hilden, Germany), and 20 μl of the purified genomic DNA (2.76 µg) was incubated with CecA or NAL for 1 h at room temperature. RNA binding was measured by incubating CecA or NAL with total RNA (13 µg) on ice for 1 h. We then added 2 μl of native loading buffer, and a 10-μl aliquot was separated by 1% agarose gel electrophoresis in 1 × Tris-borate-EDTA buffer (45 mM Tris-borate, 1 mM EDTA, pH 8.0). All gels were derived from the same experiment and were processed in parallel (Supplementary Figs 8 and 9).

The eDNA-binding assay was developed by inoculating CFT073 at OD_600_ = 0.04 in 250 µl LB medium supplemented with 0.2 µM BOBO-3 (Thermo Fisher Scientific), a non-cell-permeable cyanine-based DNA dye, which has high affinity for nucleic acids and fluoresces strongly when bound to nucleic acids but not in their absence. The bacteria were grown for 24 h in the presence of CecA, NAL, CecA + NAL, or Tris-buffered saline (control) in black, F-bottom 96-well plates (Greiner Bio-One) at 37 °C with slight orbital shaking. A well-area scan was carried out at hourly intervals for 21 h to detect eDNA fluorescence at excitation and emission wavelengths of 570 and 602 nm, respectively, in a Synergy H4 Hybrid Multi-Mode plate reader (BioTek). The readout was presented in relative fluorescence units (RFU).

CecA was predicted to bind to the WKIFKKI sequence of DnaK (http://limbo.switchlab.org). The *E. coli* gyrase ATPase activity was determined using the *E. coli* gyrase ATPase Assay Kit Plus (ProFoldin, Hudson, Massachusetts, USA). Briefly, the 30-μl reaction mixture composed of 15 μl of water, 3 μl of sample/standard at various concentrations, 3 μl of 10 × buffer, 3 μl of 10 × DNA, 3 μl of 10 × *E. coli* DNA gyrase (enzyme) or 5 µg recombinant *E. coli* DnaK protein (Abcam, Cambridge, UK) and 3 μl of 2 mM ATP. A reaction mixture without CecA/NAL was used as positive control. A reaction mixture without DNA gyrase or DnaK was used as the negative control. The reaction mixture was incubated at 37 °C (DNA gyrase ATPase activity) or 25 °C (DnaK ATPase activity) for 60 min. After incubation, 45 μl of the dye supplied with the kit was added and the absorbance was measured at 650 nm using a Synergy H4 Hybrid Multi-Mode plate reader (BioTek).

### Data analysis

Data were analyzed using GraphPad Prism v7.05 and Microsoft Excel 2013. All experiments were carried out a minimum of three times. Significant differences between pairs of values were compared using one-way analysis of variance (ANOVA) with Dunnett’s multiple comparison test, Mann–Whitney *U-*test, and Holm-Šídák test.

### Reporting summary

Further information on experimental design is available in the Nature Research Reporting Summary linked to this article.

## Supplementary information

Supplementary Information

Reporting Summary

## Data Availability

The authors declare that [the/all other] data supporting the findings of this study are available within the paper [and its supplementary information files].

## References

[1] Flores-Mireles AL, Walker JN, Caparon M, Hultgren SJ (2015). Urinary tract infections: epidemiology, mechanisms of infection and treatment options. Nat. Rev. Microbiol..

[2] Toval F (2014). Characterization of *Escherichia coli* isolates from hospital inpatients or outpatients with urinary tract infection. J. Clin. Microbiol..

[3] Koo H, Howlin RP, Stoodley P, Hall-Stoodley L (2017). Targeting microbial biofilms: current and prospective therapeutic strategies. Nat. Rev. Microbiol..

[4] Yu G, Baeder DY, Regoes RR, Rolff J (2018). Predicting drug resistance evolution: insights from antimicrobial peptides and antibiotics. Proc. Biol. Sci..

[5] Zheng Z (2017). Synergistic efficacy of *Aedes aegypti* antimicrobial peptide cecropin A2 and tetracycline against *Pseudomonas aeruginosa*. Antimicrob. Agents Chemother..

[6] Heitmueller M, Billion A, Dobrindt U, Vilcinskas A, Mukherjee K (2017). Epigenetic mechanisms regulate innate immunity against uropathogenic and commensal-like *Escherichia coli* in the surrogate insect model *Galleria mellonella*. Infect. Immun..

[7] Shin YP (2010). Antimicrobial activity of a halocidin-derived peptide resistant to attacks by proteases. Antimicrob. Agents Chemother..

[8] Yan J (2013). Two hits are better than one: membrane-active and DNA binding-related double-action mechanism of NK-18, a novel antimicrobial peptide derived from mammalian NK-lysin. Antimicrob. Agents Chemother..

[9] Marr AK, Gooderham WJ, Hancock RE (2006). Antibacterial peptides for therapeutic use: obstacles and realistic outlook. Curr. Opin. Pharmacol..

[10] Hall CW, Mah TF (2017). Molecular mechanisms of biofilm-based antibiotic resistance and tolerance in pathogenic bacteria. Fems. Microbiol. Rev..

[11] Devaraj A, Justice SS, Bakaletz LO, Goodman SD (2015). DNABII proteins play a central role in UPEC biofilm structure. Mol. Microbiol..

[12] Coldham NG, Webber M, Woodward MJ, Piddock LJ (2010). A 96-well plate fluorescence assay for assessment of cellular permeability and active efflux in *Salmonella enterica* serovar *typhimurium* and *Escherichia coli*. J. Antimicrob. Chemother..

[13] Alav I, Sutton JM, Rahman KM (2018). Role of bacterial efflux pumps in biofilm formation. J. Antimicrob. Chemother..

[14] Blair JM, Piddock LJ (2016). How to measure export via bacterial multidrug resistance efflux pumps. MBio.

[15] Nakao R, Myint SL, Wai SN, Uhlin BE (2018). Enhanced biofilm formation and membrane vesicle release by *Escherichia coli* expressing a commonly occurring plasmid gene, *kil*. Front. Microbiol..

[16] Ogata Y (1996). DnaK heat shock protein of *Escherichia coli* maintains the negative supercoiling of DNA against thermal stress. J. Biol. Chem..

[17] Orhan G, Bayram A, Zer Y, Balci I (2005). Synergy tests by E test and checkerboard methods of antimicrobial combinations against *Brucella melitensis*. J. Clin. Microbiol..

[18] Johnson MD (2004). Combination antifungal therapy. Antimicrob. Agents Chemother..

[19] Tonk M (2018). Antiplasmodial activity of tick defensins in a mouse model of malaria. Ticks Tick. Borne Dis..

[20] Luna-Ramirez K, Tonk M, Rahnamaeian M, Vilcinskas A (2017). Bioactivity of natural and engineered antimicrobial peptides from venom of the scorpions *Urodacus yaschenkoi* and *U. manicatus*. Toxins.

[21] Mukherjee K, Hain T, Fischer R, Chakraborty T, Vilcinskas A (2013). Brain infection and activation of neuronal repair mechanisms by the human pathogen *Listeria monocytogenes* in the lepidopteran model host *Galleria mellonella*. Virulence.

[22] Zdybicka-Barabas A (2013). Synergistic action of *Galleria mellonella* apolipophorin III and lysozyme against Gram-negative bacteria. Biochim. Biophys. Acta.

[23] Horcas I (2007). WSxM: a software for scanning probe microscopy and a tool for nanotechnology. Rev. Sci. Instrum..

[24] Glaeser SP (2016). Non‐pathogenic *Rhizobium radiobacter* F4 exhibits plant beneficial activity independent of its host *Piriformospora indica*. ISME J..

[25] Iyer R, Ferrari A, Rijnbrand R, Erwin AL (2015). A fluorescent microplate assay quantifies bacterial efflux and demonstrates two distinct compound binding sites in AcrB. Antimicrob. Agents Chemother..

