# Supplementary material for: The insect antimicrobial peptide cecropin A disrupts uropathogenic Escherichia coli biofilms
Source: NPJ Biofilms Microbiomes. 2020 Feb 12;6:6. doi: 10.1038/s41522-020-0116-3 (PMC7016129; doi:10.1038/s41522-020-0116-3)
Supplement: Supplementary file 1 — Supplementary Information [file 41522_2020_116_MOESM1_ESM.pdf]

The insect antimicrobial peptide cecropin A disrupts uropathogenic *Escherichia coli* biofilms

Miriam Kalsy[1], Miray Tonk[2,3], Martin Hardt[4], Ulrich Dobrindt[5], Agnieszka Zdybicka-Barabas[6], Malgorzata Cytrynska[6], Andreas Vilcinskas[1,2,3*], Krishnendu Mukherjee[1,5*]

[1]Fraunhofer Institute for Molecular Biology and Applied Ecology, Department of Bioresources, Giessen 35394, Germany

[2]Institute for Insect Biotechnology, Justus Liebig University, Giessen 35392, Germany

[3]LOEWE Centre for Translational Biodiversity Genomics (LOEWE-TBG), Frankfurt 60325, Germany

[4]Imaging Unit, Biomedical Research Center Seltersberg (BFS), Justus Liebig University, Giessen 35392, Germany

[5]Institute of Hygiene, University of Muenster, Muenster 48149, Germany

[6]Department of Immunobiology, Institute of Biological Sciences, Faculty of Biology and Biotechnology, Maria Curie-Skłodowska University, Lublin, Poland

M. K. and K.M. contributed equally to this work

*Correspondence to Krishnendu.Mukherjee@agrar.uni-giessen.de, Krishnendu.Mukherjee@ukmuenster.de, Andreas.Vilcinskas@agrar.uni-giessen.de

Supplementary Information

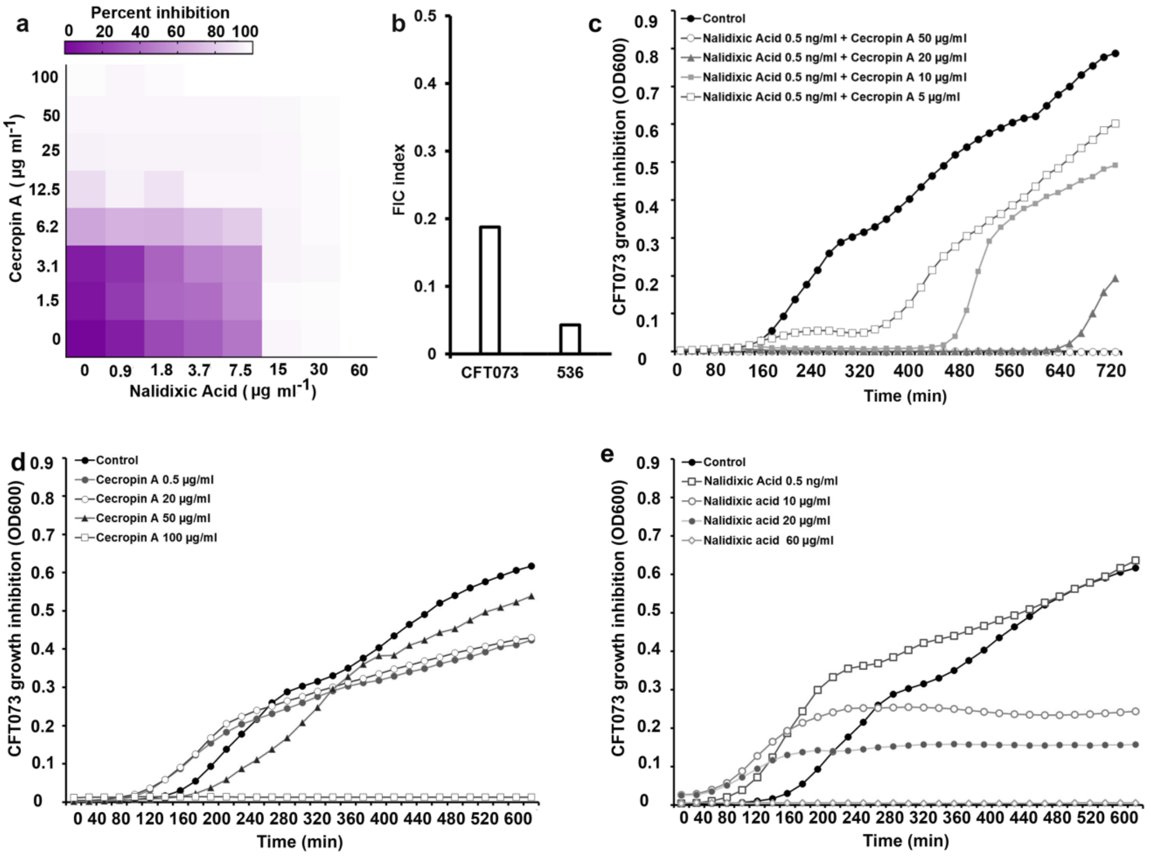

Supplementary **Figure 1. Synergy between cecropin A (CecA) and nalidixic acid (NAL).** (a) Heat plot showing synergistic growth inhibition of uropathogenic *Escherichia coli* (UPEC) strain CFT073 by the combination of CecA + NAL. The minimum inhibitory concentration (MICs) for CecA and NAL were 100 µg/ml and 60 µg/ml respectively. (b) The fractional inhibitory concentration (FIC) index was determined for the UPEC strains CFT073 and 536. Dose-dependent survival of the UPEC strain CFT073 after inoculation with CecA, NAL, and CecA + NAL. Bacteria were cultured in LB supplemented with (c) 0.5 ng/ml NAL in combination with 5 µg/ml, 10 µg/ml, 20 µg/ml, 50 µg/ml CecA, (d) 0.5 µg/ml, 20 µg/ml, 50 µg/ml, 100 µg/ml CecA, and (e) 0.5 ng/ml, 10 µg/ml, 20 µg/ml, 60 µg/ml NAL the optical density at 600 nm (OD600) was measured every 40 mins. The experiments were carried out three times with similar results.

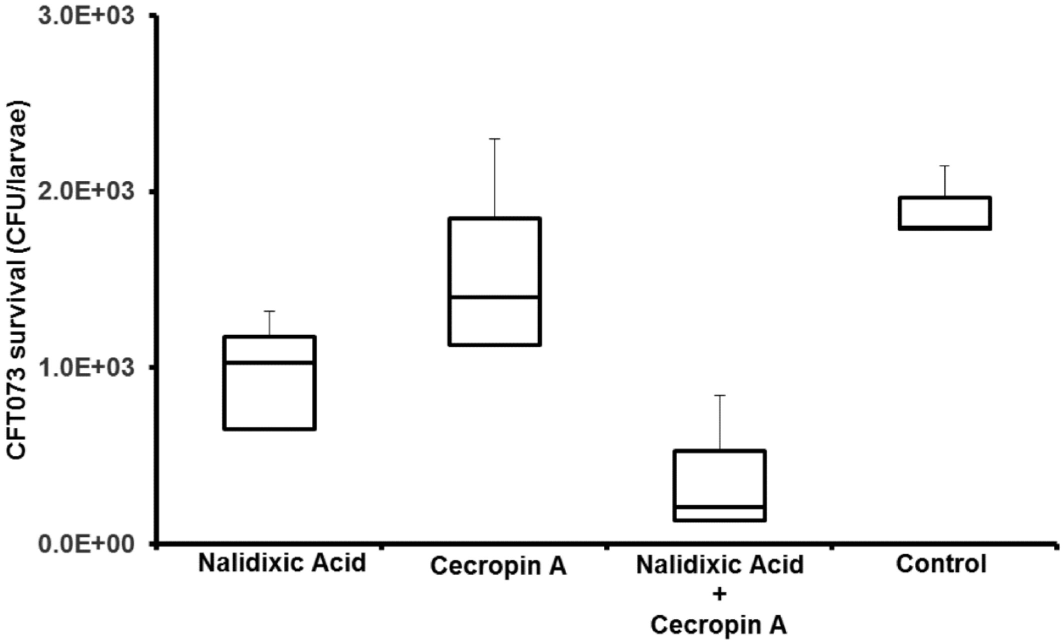

Supplementary **Figure 2. Survival of uropathogenic *Escherichia coli* in *Galleria mellonella* larvae after treatment with cecropin A (CecA) and nalidixic acid (NAL).** To determine the bacterial survival rate, we measured the *E. coli* load in the infected larvae 24 h after treatment with NAL (0.5 ng/ml), CecA (50 µg/ml), or their combination. Homogenates of 10 larvae were plated individually on *E. coli* selective "*E. coli* direct" (ECD) agar plates. The results are shown as a box-whisker plot, with the box representing values from the lower to the upper quartile and the whiskers representing the range. Each experiment was carried out three times with 10 larvae per treatment.

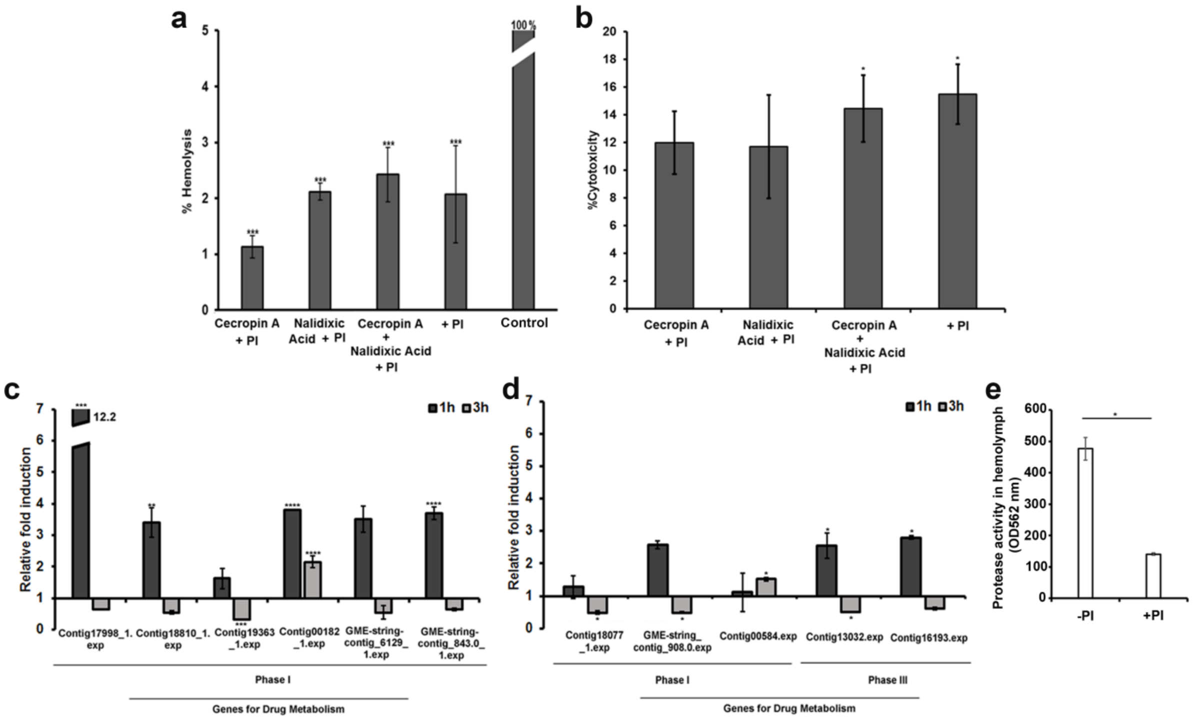

Supplementary **Figure 3. Influence of protease inhibitor on cytotoxicity and expression of genes related to drug metabolism following cecropin A (CecA) and nalidixic acid (NAL) treatment.** (a) Porcine erythrocytes were treated with cecropin A (CecA; 50 µg/ml), nalidixic acid (NAL; 0.5 ng/ml), their combination (CecA + NAL) in presence of protease inhibitor (PI) in comparison to 10% Triton X-100 to determine hemolysis. (b) The viability of BHK-21 fibroblast cells was measured after treatment with CecA (50 µg/ml), NAL (0.5 ng/ml), their combination (CecA + NAL) in presence of PI in comparison to untreated control. (c-d) Steady state mRNA levels of putative marker genes related to drug metabolism phases I and III in CFT073-infected larvae 1 and 3 h after treatment with a combination of CecA (50 µg/ml) and NAL (0.5 ng/ml) in the presence and absence of PI. Expression levels were determined by quantitative real-time RT-PCR and the significance of induction is shown relative to CFT073-infected larvae 1 and 3 h after treatment with the combination of CecA (50 µg/ml) and NAL (0.5 ng/ml) in the absence of PI. The selected expressed sequence tags from Table S1 comprise (c) Contig 17998_1.exp, Contig 18810_1.exp, Contig 19363_1.exp, Contig 00182_1.exp, GME-string-contig_6129_1.exp, and GME-string-contig_843.0_1.exp; and (d)

Contig 18077_1.exp, GME-string_contig_908.0.exp, Contig 00584.exp, Contig 13032.exp, and Contig 16193.exp. Values were normalized against expression levels of the housekeeping gene 18*S* rRNA. (e) Protease activity of *G. mellonella* hemolymph with and without the administration of PI. Values are means and standard errors: n = 3 (panels a-e) (**P* < 0.05; **P < 0.005; ****P* < 0.0005; *****P* < 0.0001; (a-b) One Way Anova, Dunnett's multiple comparison test, (c-e) Holm-Šídák correction).

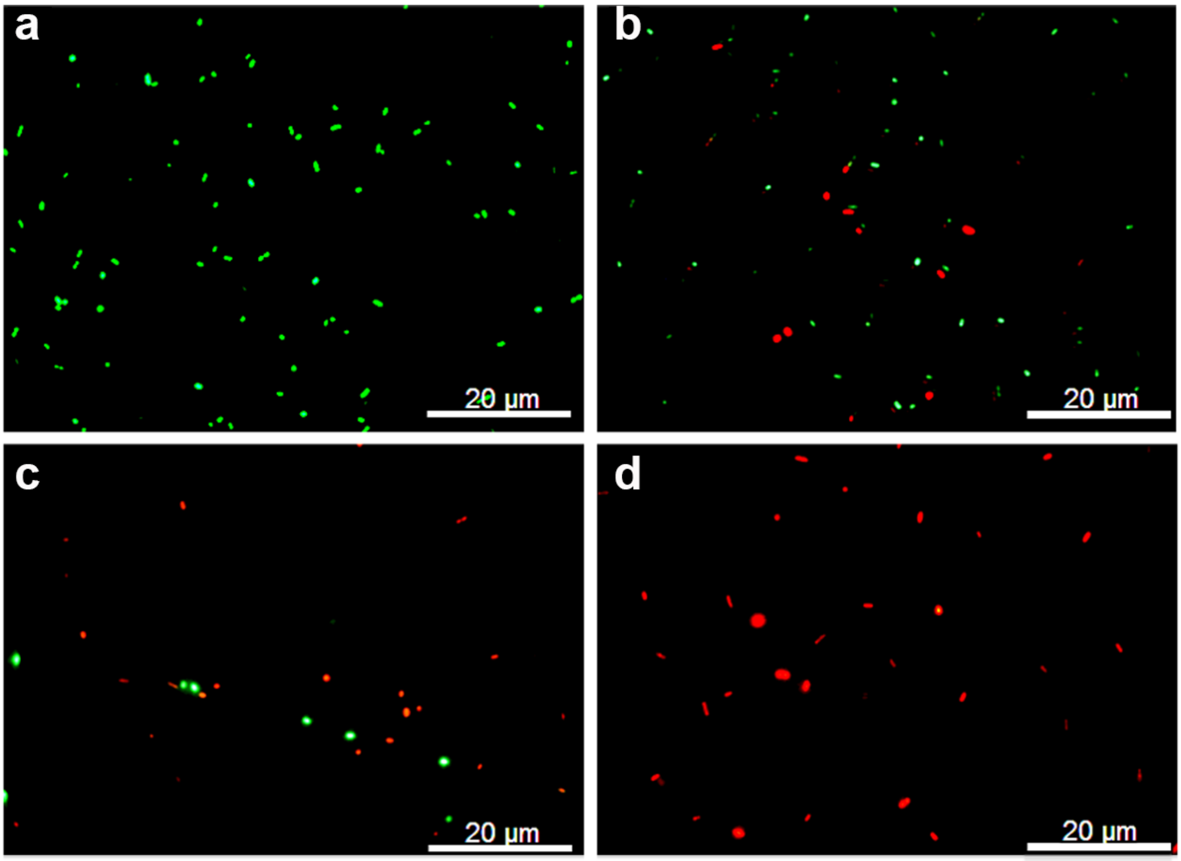

Supplementary **Figure 4. Exposure of uropathogenic *Escherichia coli* (UPEC) to cecropin A (CecA) and/or nalidixic acid (NAL)**. Fluorescence microscopy showing UPEC strain CFT073 exposed to CecA and/or NAL. Bacteria were grown at 37°C in the presence of (a) dimethylsulfoxide, (b) NAL (0.5 ng/ml), (c) CecA (50 µg/ml), or (d) a combination of CecA (50 µg/ml) and NAL (0.5 ng/ml). Bacteria were stained with SYTO 9, which shows live cells

as green, and propidium iodide, which stains dead cells red. The experiment was carried out three times with similar results.

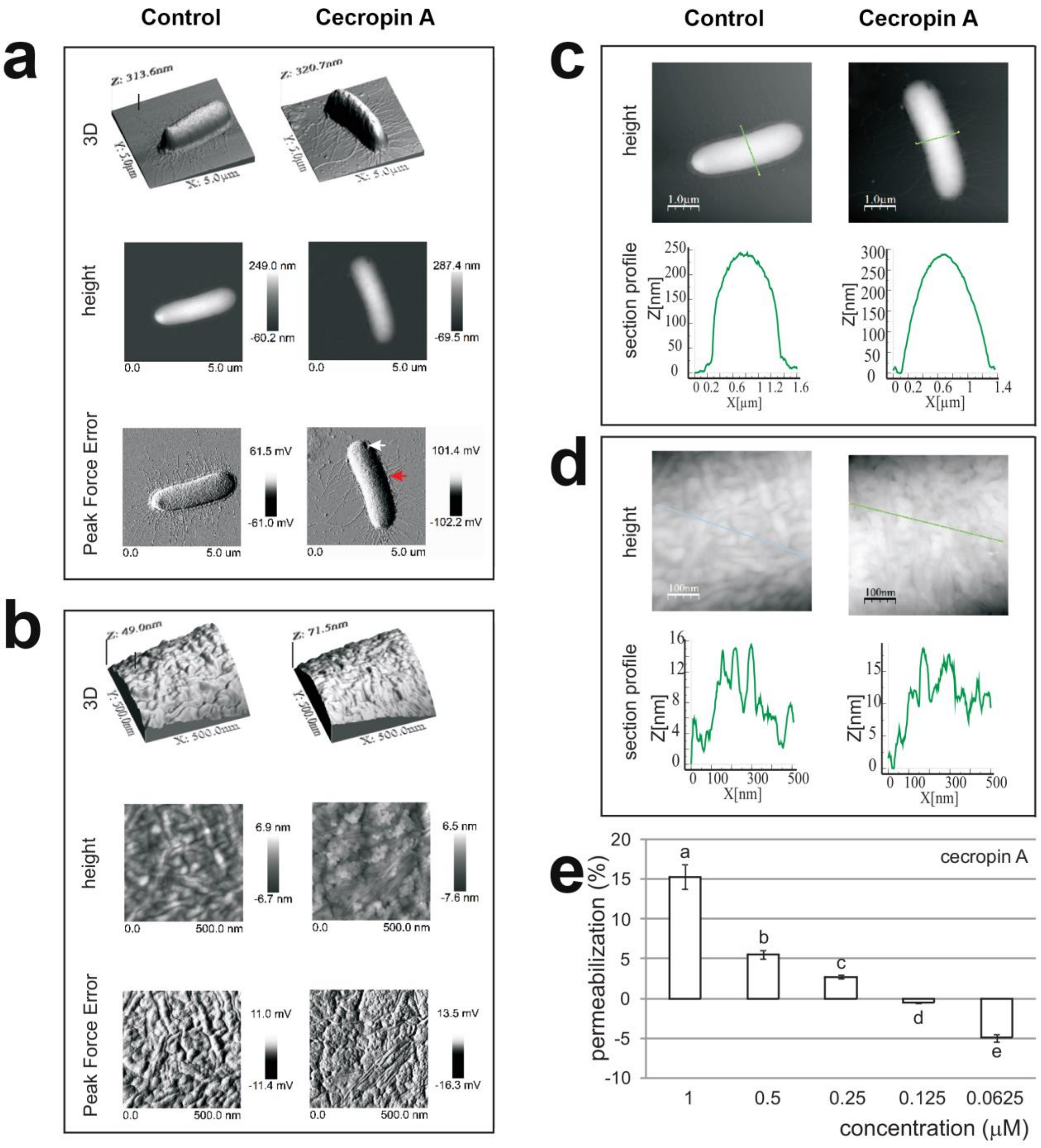

Supplementary **Figure 5. Interactions of cecropin A with *E. coli* JM83 cell envelope.** The bacteria were incubated without (control) or in the presence of CecA (0.25µM) and then imaged by AFM. (a-b) **Bacterial surface alterations.** Three dimensional (3D), height, and peak force error 5µm×5µm (a) and 500nm×500nm (b) images of the bacteria are presented. In

the peak force error and height images the white arrows indicate recesses observed after treatment of the bacteria with CecA. The red arrows in the peak force error image (a) indicate damaged envelope. (c-d) **Section profiles of bacterial cell surface.** The height 5µm×5µm (c) and 500nm×500nm (d) images of the bacterial cell surface. The bottom panels demonstrate the section profiles corresponding to the lines marked in the upper panels. The bars represent 1µm (upper panels) and 100nm (bottom panels). (e) **Bacterial membrane permeabilization.** The bacteria were incubated without or in the presence of CecA (a) for 45 min at 37°C. Then permeabilization of the membrane was estimated by β-galactosidase assay. Live bacteria incubated alone and dead bacteria after treatment with 5µM synthetic cecropin B served as the control samples. The permeabilization level of the dead bacteria was assumed as 100%. Data represent means and standard deviations: n = 6 (panel a-e). Statistically significant differences are indicated with different letters ($P \leq 0.001$; One-Way Anova).

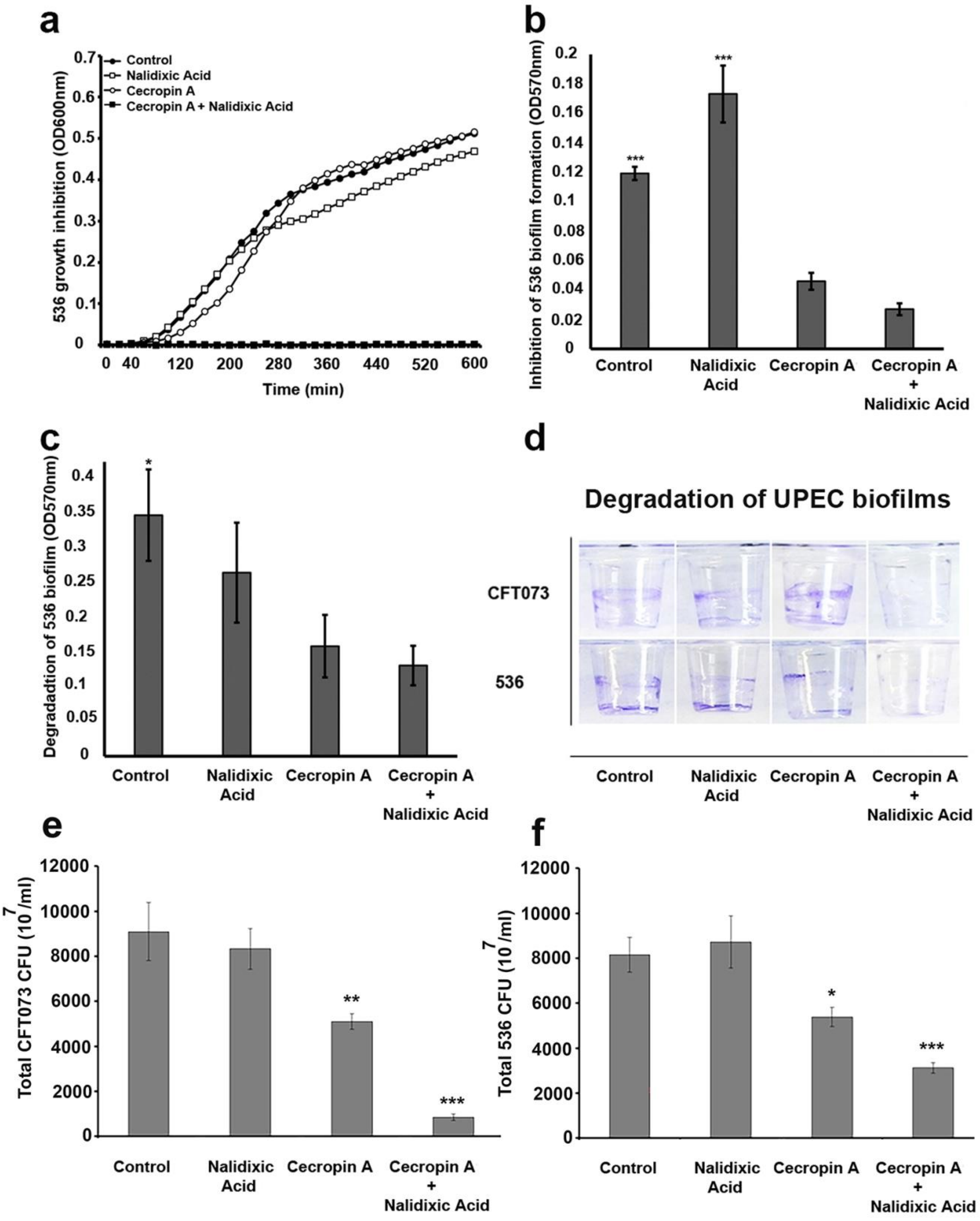

Supplementary **Figure 6. Effect of synergy between cecropin A (CecA) and/ nalidixic acid (NAL) on planktonic and biofilm forming uropathogenic *Escherichia coli* (UPEC) cells.** (a) Time-kill curves of CecA (50 μg/ml), NAL (0.5 ng/ml), and the CecA (50 μg/ml) + NAL (0.5 ng/ml) combination against UPEC strain 536. (b) Percentage inhibition of 536 biofilms grown in LB medium supplemented with NAL (0.5 ng/ml) and CecA (50 μg/ml) or their

combination for 48 h (***$P < 0.0005$ - compared with CecA + NAL). (c) Percentage degradation of 536 biofilms grown in LB medium for the first 24 h without any treatment and for the second 24 h with NAL (0.5 ng/ml), CecA (50 µg/ml) or their combination (*$P < 0.05$ - compared with CecA + NAL). (d) Degradation of CFT073 and 536 biofilms in the presence or absence of NAL (0.5 ng/ml), CecA (50 µg/ml), or their combination. (e-f) Quantification of CFT073 and 536 biofilms by plate count (CFU) following 24 h of growth in presence of NAL (0.5 ng/ml), CecA (50 µg/ml), or their combination. Biofilm counts are expressed as means of CFU per ml (*$P < 0.05$; **$P < 0.005$; ***$P < 0.0005$ - compared with control). Values are means and standard errors: n = 4 (panels b, c, e, f) (One-Way Anova, Holm-Šídák correction).

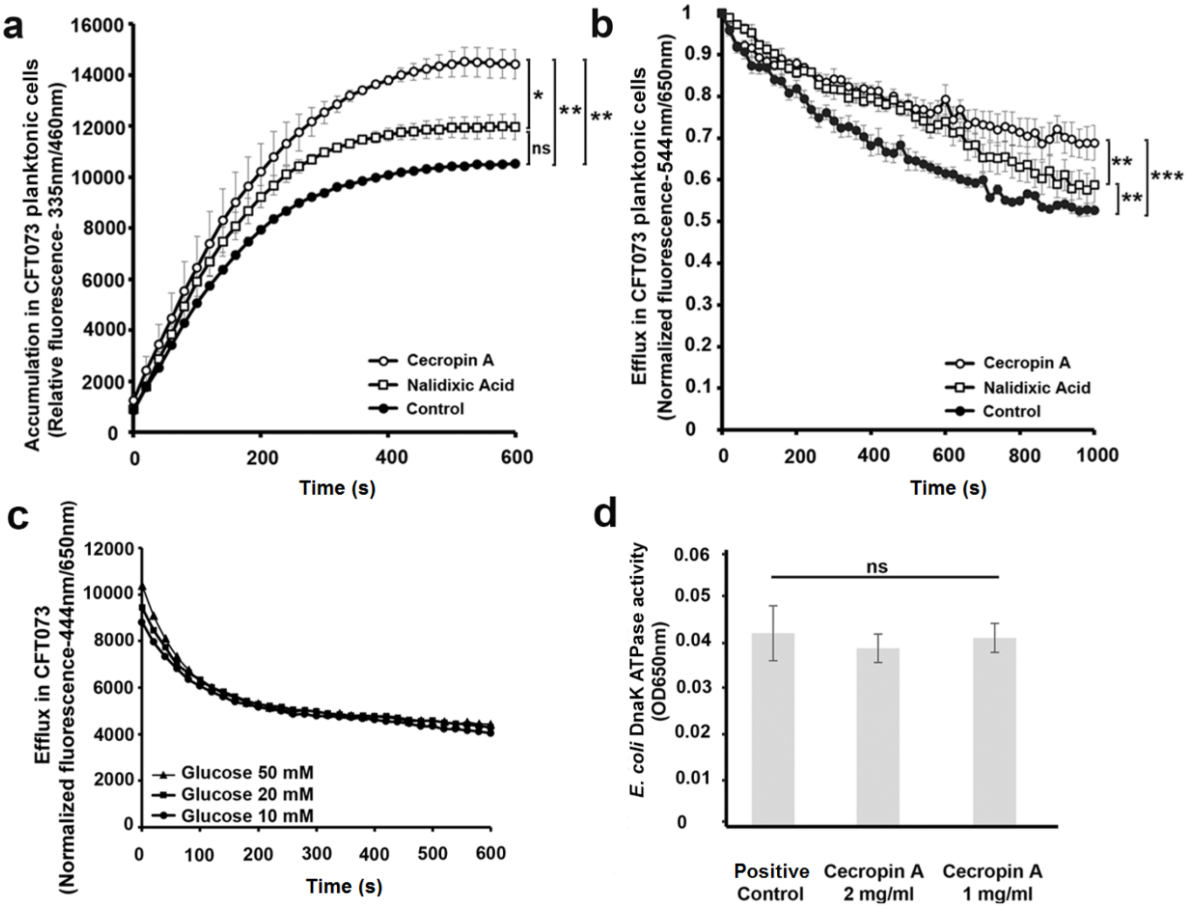

Supplementary **Figure 7. Cecropin A (CecA) accumulation and efflux by planktonic uropathogenic *Escherichia coli* (UPEC) cells, and inhibition of DnaK ATPase activity.** (a) Steady-state levels of H33342 (2.5 µM) accumulating in CFT073 planktonic cells with and without exposure to CecA (10 µg/ml) or nalidixic acid (NAL; 0.5 ng/ml). (b) Inhibition of Nile red efflux by CecA (10 µg/ml) and NAL (0.5 ng/ml). Efflux was triggered at 100 s by the addition of 20 mM glucose. The intensity of fluorescence emission from Nile red was presented to show the effects of CecA, NAL or the absence of treatment in CFT073 planktonic cells. (c) The intensity of fluorescence emission from Nile red is presented to show the degree of efflux triggered by different concentrations of glucose. (d) Inhibition of DnaK ATPase activity by CecA. Values are means and standard errors: n = 3 (panels a-b, d). Significance was determined by one-way ANOVA and Holm-Šídák correction, (*P < 0.05; **P < 0.005; ***P < 0.0005; ns – not significant).

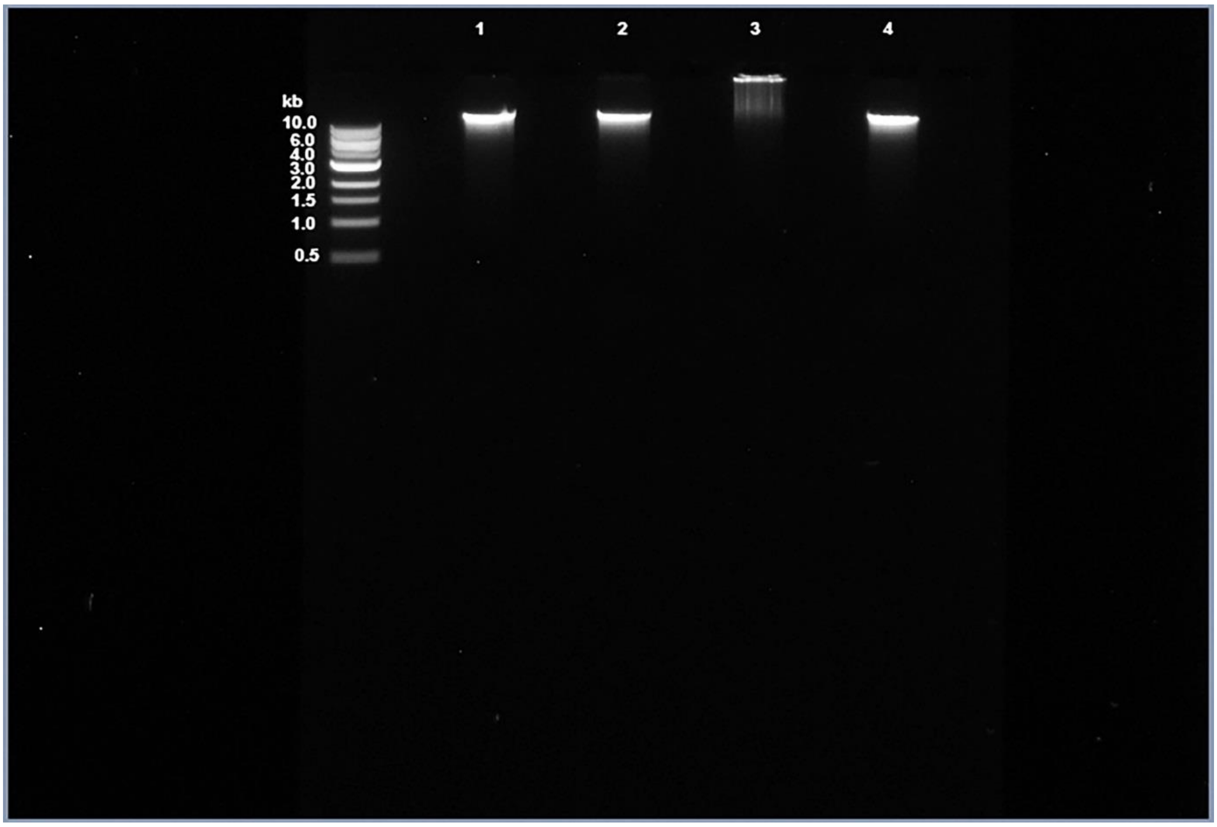

Supplementary **Figure 8. Interaction of cecropin A (CecA) and nalidixic acid (NAL) with uropathogenic *Escherichia coli* (UPEC) DNA**. Full, uncropped image of Fig. 2e showing decreasing intensities of DNA bands from CFT073 cells treated with high concentrations of CecA were visualized by gel electrophoresis to highlight the DNA binding activity of CecA. Lanes 1-4 represent untreated DNA sample and DNA treated with CecA 125 µM, CecA 250 µM, and NAL 25 µM respectively. Marker represents 1 kb DNA ladder.

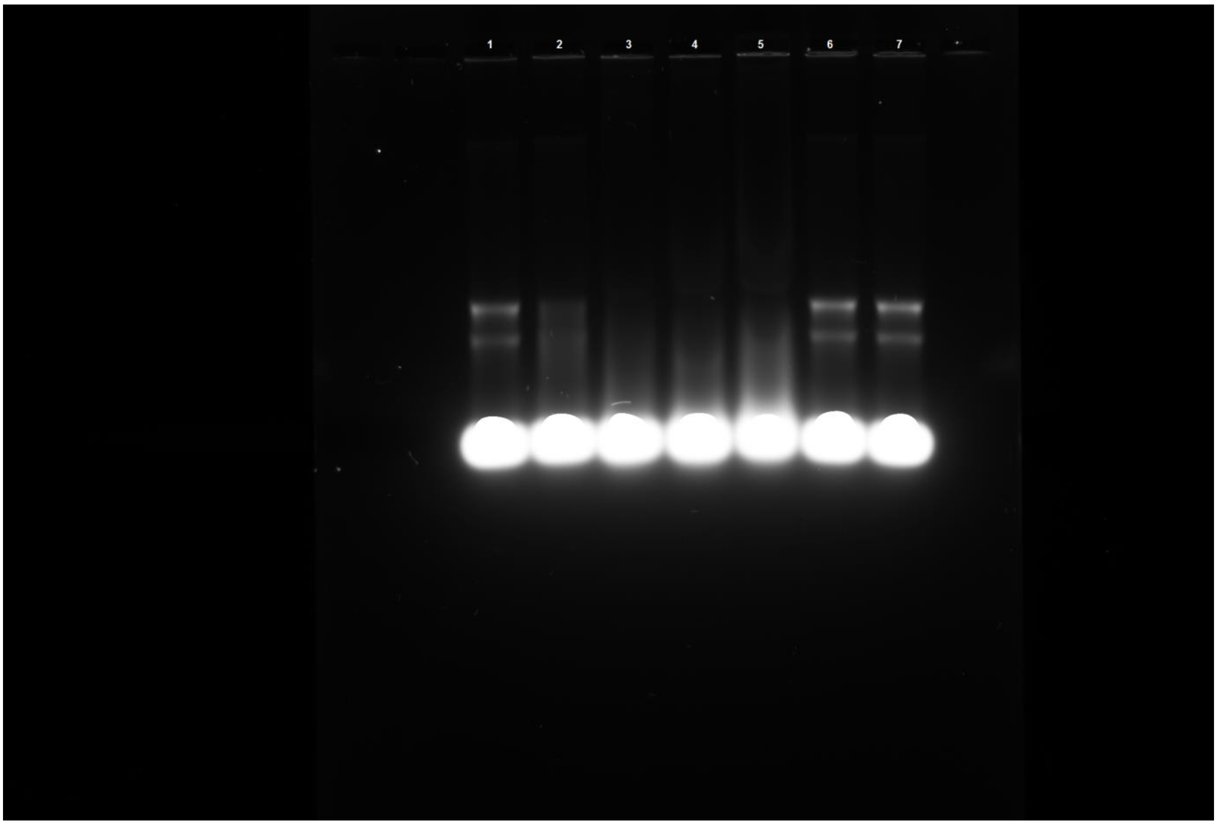

Supplementary **Figure 9. Interaction of cecropin A (CecA) and nalidixic acid (NAL) with uropathogenic *Escherichia coli* (UPEC) RNA**. Full, uncropped image of Fig. 2f showing decreasing intensities of RNA bands from CFT073 cells treated with a high concentrations of CecA were visualized by gel electrophoresis to highlight the RNA binding activity of CecA. Lanes 1-7 represent untreated RNA sample, and RNA treated with CecA 12 µM, CecA 125 µM, CecA 250 µM, CecA 375 µM, NAL 25 µM and NAL 250 µM respectively.

Supplementary **Table 1. GO terms of metabolic genes in *G. mellonella***

	**Name**	**Function**	**Process**
contig17998_1.exp	Cytochrome P450 monooxygenase	heme binding, monooxygenase activity	
contig18810_1.exp	Cytochrome P450	iron ion binding, heme binding	electron transport
contig19636_1.exp	Cytochrome P450	heme binding, monooxygenase activity	electron transport
contig00182_1.exp	Alcohol dehydrogenase	oxidoreductase activity	
GME-string_Contig_6129_1.exp	NADH-ubiquinone oxidoreductase	NADH dehydrogenase (ubiquinone) activity	mitochondrial electron transport, ubiquinone biosynthetic process
GME-string_Contig_843.0_1.exp	NADH-ubiquinone oxidoreductase	NADH dehydrogenase (ubiquinone) activity	mitochondrial electron transport, ubiquinone biosynthetic process

contig18077_1.exp	Carboxylic ester hydrolase	hydrolase activity	
GME-string_Contig_908.0.exp	Epoxide hydrolase	cis-stilbene-oxide hydrolase activity, epoxide hydrolase activity	response to toxin, aromatic compound catabolic process, xenobiotic metabolic
contig00548.exp	Epoxide hydrolase	epoxide hydrolase activity	Aromatic compound catabolic process, xenobiotic metabolic
contig13032_1.exp	ATP-binding cassette sub-family B member 3	ATPase activity,	Transport
contig16193_1.exp	ATP-binding cassette sub-family B member 6	ATPase activity	

Supplementary **Table 2. The effect of *G. mellonella* cecropin A on nanomechanical properties of *E. coli* JM83 cell surface**. The bacteria were incubated without (control) or in the presence of cecropin (0.25µM) and then analyzed by AFM. The results are presented as ±SD. The same letters indicate statistically significant differences between the peptide-treated experimental groups (Mann-Whitney U test).

	**control**	**Cecropin A (0.25µM)**
Roughness [nm]	1.252 (± 0.259)	1.310 (± 0.32)[b]
Young's modulus [MPa]	2468 (± 330.9)	2249.4 (± 602.5)
Adhesion forces [nN]	0.1383 (± 0.099)	0.1511 (± 0.087)

Supplementary **Table 3. DNA binding prediction with DP-bind (http://lcg.rit.albany.edu/dp-bind)**

**Pos.**	**Res**	**S_LBL**	**S_PRB**	**K_LBL**	**K_PRB**	**P_LBL**	**P_PRB**	**MAJ_CON**	**STR_Con**
**1**	K	1	0.7438	1	0.8041	1	0.6920	1	1

**2**	W	1	0.6445	1	0.7472	1	0.5916	1	1
**3**	K	1	0.8500	1	0.8064	1	0.7230	1	1
**4**	I	1	0.5400	1	0.5641	0	0.6078	1	NA
**5**	F	1	0.5694	0	0.5285	0	0.5584	0	NA
**6**	K	0	0.6832	0	0.7319	0	0.6488	0	0
**7**	K	0	0.7866	0	0.6343	0	0.7673	0	0
**8**	I	0	0.8808	0	0.9527	0	0.7976	0	0
**9**	E	0	0.7475	0	0.8435	0	0.8544	0	0
**10**	K	0	0.5473	0	0.5782	0	0.5629	0	0
**11**	A	0	0.8299	0	0.8529	0	0.8222	0	0
**12**	G	1	0.5501	0	0.5263	0	0.6405	0	NA
**13**	R	1	0.6461	1	0.7525	1	0.6948	1	1
**14**	N	0	0.5480	0	0.5187	1	0.5692	0	NA
**15**	I	1	0.5373	1	0.5966	0	0.5382	1	NA
**16**	R	1	0.8963	1	0.9053	1	0.7335	1	1
**17**	D	1	0.5953	0	0.6283	0	0.7089	0	NA
**18**	G	0	0.6696	0	0.6556	0	0.7081	0	0

**19**	I	0	0.8476	0	0.7282	0	0.7451	0	0
**20**	I	0	0.8027	0	0.8527	0	0.7220	0	0
**21**	K	1	0.8680	1	0.7283	1	0.7961	1	1
**22**	A	1	0.5636	1	0.6413	1	0.5415	1	1
**23**	G	1	0.7206	1	0.7784	0	0.5623	1	NA
**24**	P	1	0.7475	1	0.6821	1	0.5845	1	1
**25**	A	1	0.6938	0	0.6882	0	0.5410	1	NA
**26**	V	0	7214	0	0.7736	0	0.7546	0	0
**27**	S	0	0..623 8	0	0.8814	0	0.8395	0	0
**28**	V	0	0.9167	0	0.9701	0	0.8793	0	0
**29**	V	0	0.7434	0	0.8666	0	0.7770	0	0
**30**	G	0	0.6768	0	0.5626	1	0.5217	0	NA
**31**	E	0	0.5838	0	0.7517	0	0.5652	0	0
**32**	A	0	0.6251	0	0.6017	0	0.7079	0	0
**33**	A	1	0.7945	1	0.5359	1	0.6842	1	1
**34**	T	1	0.7102	1	0.5652	0	0.5739	1	NA

**35**	I	0	0.5975	0	0.6333	0	0.6706	0	0
**36**	Y	1	0.5782	1	0.6687	1	0.5869	1	1
**37**	K	1	0.8050	1	0.7539	1	0.6779	1	1
**38**	T	1	0.7461	1	0.5952	1	0.5329	1	1
**39**	G	1	0.7858	1	0.7370	1	0.5112	1	1

Supplementary **Table 4**. **RNA binding prediction with FastRNABindR (http://ailab.ist.psu.edu/FastRNABindR/)**

**Pos**	**Residue**	**Predicted score**	**Predicted labels**
**1**	K	0.72	1
**2**	W	0.73	1
**3**	K	0.73	1
**4**	I	0.45	0
**5**	F	0.58	0
**6**	K	0.73	1
**7**	K	0.7	1

**8**	I	0.26	0
**9**	E	0.55	0
**10**	K	0.75	1
**11**	A	0.6	0
**12**	G	0.54	0
**13**	R	0.7	1
**14**	N	0.71	1
**15**	I	0.39	0
**16**	R	0.66	1
**17**	D	0.44	0
**18**	G	0.44	0
**19**	I	0.35	0
**20**	I	0.3	0
**21**	K	0.63	0
**22**	A	0.46	0
**23**	G	0.42	0
**24**	P	0.47	0

**25**	A	0.4	0
**26**	V	0.45,	0
**27**	S	0.33	0
**28**	V	0.37	0
**29**	V	0.25	0
**30**	G	,0.44	0
**31**	E	0.6	0
**32**	A	0.29	0
**33**	A	0.49	0
**34**	T	0.55	0
**35**	I	0.31	0
**36**	Y	0.38	0
**37**	K	0.7	1
**38**	T	0.58	0
**39**	G	0.43	0

Supplementary **Table 5**. **Primer Sequences**

	**Forward**	**Reverse**
contig17998_1.exp	CCAACGTGACGTAGATGTGG	TCAGATGGAAGACGGTGACA
contig18810_1.exp	CCACGACGGACAATTGACTT	TCCGGATCGAATACTTCTGG
contig19636_1.exp	CAGCCATTGCATAATCATATCC	CGTTCATGCCCGTATTTAATG
contig00182_1.exp	TTTGCCAGCAGAACTCAATG	CAAGGTATACCGCAGGAGGA
GME-string_Contig_6129_1.exp	GATCTGATGCCGGAACCTAA	TGAAGCATGCCGTATAGCAG
GME-string_Contig_843.0_1.exp	TCCCGGTACGGTACTTTCAG	ATCGTCAAGTCGTGCAACAG
contig18077_1.exp	ACGGTGGAACAATCCTTGAG	GTAGCTCCCGGTAACAATGG
GME-string_Contig_908.0.exp	GTGCCTTTACTGGCCATCAT	TCTTCAAGCGGGTCTTGAGT

contig00548.exp	ACCAGTGACGGGAATACAGC	GGACTTGCTGCTCCTCAAAC
contig13032_1.exp	CCAAAGGGCTACGACACAAA	TAACAGCCCGTTCACTGTTG
contig16193_1.exp	CACCCTGCAGGAACTTGAAT	TTAGCAAACTGCGGACACTG
18*S* rRNA	CACATCCAAGGAAGGCAG	AGTGTACTCATTCCGATTACGA
